# Nanotechnology opens up a new universe of nanoart

**DOI:** 10.1093/nsr/nwaf468

**Published:** 2025-11-04

**Authors:** Jinsai Tian, Jiayi Chen, Yue Liu, Baokang Niu, Junhui Chen, Mengyu Yan, Kui Su

**Affiliations:** State Key Laboratory of Advanced Technology for Materials Synthesis and Processing, Wuhan University of Technology, China; State Key Laboratory of Advanced Technology for Materials Synthesis and Processing, Wuhan University of Technology, China; State Key Laboratory of Advanced Technology for Materials Synthesis and Processing, Wuhan University of Technology, China; State Key Laboratory of Advanced Technology for Materials Synthesis and Processing, Wuhan University of Technology, China; State Key Laboratory of Advanced Technology for Materials Synthesis and Processing, Wuhan University of Technology, China; State Key Laboratory of Advanced Technology for Materials Synthesis and Processing, Wuhan University of Technology, China; International Students Office, Fudan University, China

## Abstract

Nanotechnology, bridging science and art, empowering new forms of nanoart through innovative approaches.

Nanotechnology refers to the study of the properties and interactions of matter, and the use of these unique properties to conduct multidisciplinary research. At the nanoscale, matter exhibits ‘unconventional’ and exotic phenomena. In the early 1980s, the invention of scanning tunneling microscopes (STMs) and atomic force microscopes (AFMs) unveiled the mystery of the nanoscopic world and greatly promoted nanotechnology. One famous example is the discovery of fullerenes at Rice University in 1985, in which 60 carbon atoms form a delicate sphere structure [1] (Fig. 1a). Fullerenes won the Nobel prize for chemistry in 1996 and are still among the most studied nanomaterials. In 1989, Don Eigler and Erhard Schweizer, two IBM researchers, used a scanning tunneling microscope in San Jose, California, to manipulate 35 xenon atoms to form the three capital letters ‘IBM’, demonstrating the operable properties of atoms (Fig. 1b). Different from intentional artistic creation, scientific nano-imaging demo is more inclined to scientific research and verification of technical principles. The other significant progress in nanomaterials is the synthesis of carbon nanotubes. In 1991, Japanese scientist Sumio Iijima produced carbon nanotubes using an arc method [2] (Fig. 1c). It was demonstrated as the strongest and stiffest material at that time.

**Figure 1.**
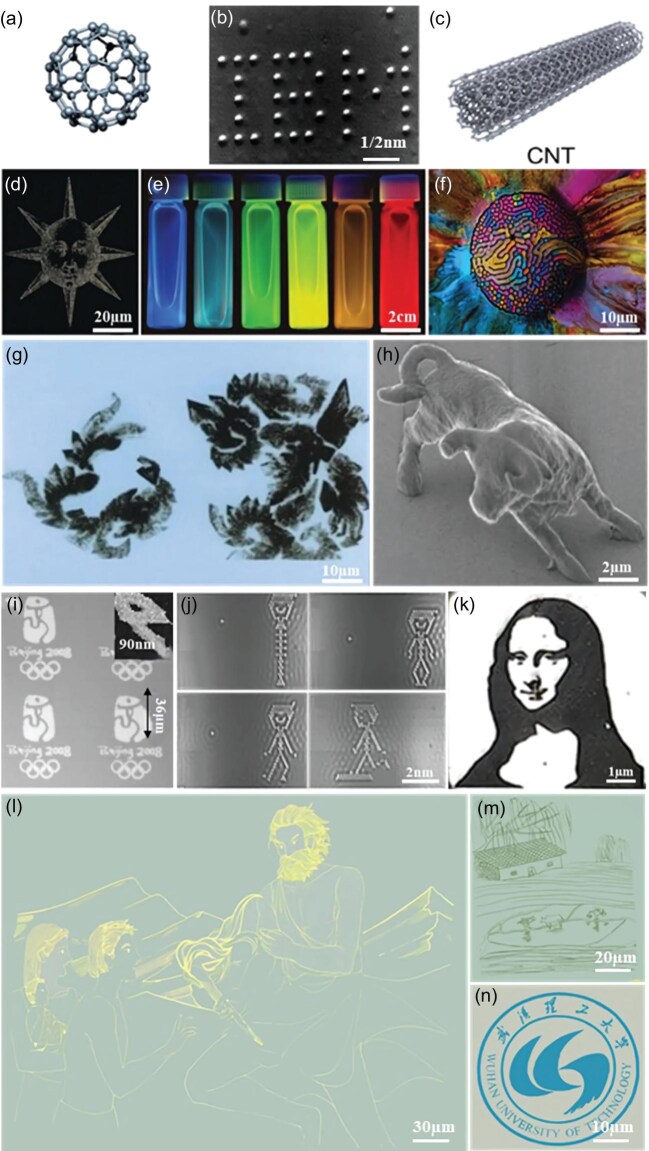
Nanoart pieces created through diverse nanoscience and nanotechnology techniques. (a) The fullerene model. Reproduced from ref. [1] with permission from the American Chemical Society, copyright 2018. (b) Nanoscale IBM logo fabricated with 35 xenon atoms in a scanning tunneling microscope. Reproduced with permission from IBM Corporation, copyright 2012. (c) Carbon nanotube model. Reproduced from ref. [2] with permission from the American Chemical Society, copyright 2024. (d) Nano gold particles ‘printed’ the sun. Reproduced from ref. [3] with permission from Springer Nature, copyright 2007. (e) Luminescent CdSe/Zinc sulfide nanocrystals. Reproduced from ref. [4] with permission from the Royal Society of Chemistry, copyright 2007. (f) Ferrofluid and watercolor created the thousand flower glass ‘Millefiori’. Reproduced from ref. [5] with permission from John Wiley and Sons. copyright 2015. (g) Aggregation and assembly of silver nanoparticles into ‘wildflowers’ and ‘seahorses’. (h) A three-dimensional bull statue of the size of red blood cells. Reproduced from ref. [6] with permission from Springer Nature, copyright 2001. (i) Nano badges printed by microcontact printing. Reproduced from ref. [7] with permission from Springer Nature, copyright 2009. (j) Plane 2D art under STM manipulation. Reproduced with permission from IBM Corporation, copyright 2012. (k) Mona Lisa pattern processed by focused ion beam. (l–n) Color image photos are deposited by multi-step patterning of nanofilms.

The world’s earliest nanoart works were created by scientists in the laboratory. For example, nanoparticles can be printed as ‘ink’ to create visually appealing images. Inspired by this, scientists used gold nanoparticles as ‘ink’ to ‘print’ the sun [3] (Fig. 1d). Figure 1e shows a series of vials containing luminescent CdSe/ZnS nanocrystals (diameter = 2.3–5.5 nm), that emit light at 470, 480, 520, 560, 594, and 620 nm (left to right), producing colorful visual art [4]. Ferrofluid can also be applied to create attractive art. Fabian Oefner, created the millefiori glass ‘Millefiori’ using ferrofluid and watercolor (Fig. 1f). Professor Wu Quande, the director of the Center for Nanoscience and Nanotechnology at Peking University, has published a number of electron microscope photos starting from 1979, among which the images of ‘wildflowers’ and ‘seahorses’ assembled by silver nanoparticles have the highest aesthetic value [5] (Fig. 1g). These artworks constitute the earliest known nanoart creations. In 2001, physicists at Osaka University used a laser to carve a three-dimensional bull statue with the size of a red blood cell (Fig. 1h), which is probably the earliest nanosculpture [6].

After entering the 21st century, with the rapid development of science and technology, many nanotechnologies have gradually become mature. These include scanning probe microscopy (SPM) techniques, nano-imprinting, nano-printing, CVD-induced growth, chemical self-assembly, nano-optics or micro-droplet freezing, and other methods. Through these nanotechnologies, the desired nano-images can be produced in a controllable, regular, and even aesthetic way. For instance, researchers successfully utilized nanoprinting technology to create the emblem for the 2008 Beijing Olympics [7] (Fig. 1i). This printing method breaks through the limit of traditional printing techniques that can only print ink dots larger than 10 microns, and enables scientists to accurately place individual particles according to their own intentions. This technology can significantly decrease the production costs of nanodevices, and promote the application of nanoscale biosensors and future computer chips. In 2013, IBM scientists used SPM to successfully manipulate carbon monoxide molecules and recorded the world’s smallest movie ‘The Boy and His Atom’ [8] (Fig. 1j). In addition, using ion beams for precise cutting and deposition, focused ion beam (FIB) etching patterns exhibit unique artistic beauty (Fig. 1k). As the design pattern is at the nanoscale, science and technology such as electron microscopes need to be used as an aid, which well reflects the integration of science and art design. At the same time, the viewer can feel more surprise and novelty in appreciation due to the different angles of observation and can see rich artistic creations at a very tiny scale. This contrast has a strong visual impact.

Nanotechnology has brought major innovations in concepts, thinking, and methods to nanoart, enabling designs that are invisible in the macroscopic world. Scientists and researchers can use various methods to concisely disseminate their research results to the public, helping people gradually accept new nanoscale technologies and products. However, nanoart often depends on nanotechnologies in laboratories, making it difficult to achieve in actual nanoart design. The high cost of nano preparation processes and related equipment, as well as the high threshold of nanotechnology, result in a scarcity of talents with interdisciplinary thinking. Therefore, developing a low-cost nanoart creation method that allows more people to recognize and participate in nanoart creation has become one of the most critical challenges at present.

Ultraviolet (UV) lithography technology, with its relatively simple operational process and low equipment cost, has become an effective method for nanoscale pattern production. By precisely controlling the exposure time and intensity of UV light, artists can create fine nanoscale patterns with photoresist. This process is not only cost-effective, but also enables high-precision replication of patterns, providing the possibility for large-scale nanoart creation. Meanwhile, using magnetron sputtering technology, it is possible to uniformly deposit nano-thin films onto the substrate. When sunlight shines on the prepared nanofilms, an interference phenomenon occurs [9]. This is able to control the nanofilm color by varying its thickness and the sputtering source on the substrates. By combining UV lithography technology, artists can further create nanoart works with unique visual effects by patterning thin films on substrates deposited with nanofilms.

We have compiled metal chromium films of different thicknesses (Fig. S2a and b), varying the magnetron sputtering time from 50 to 300 s. A series of these films were prepared, and their thicknesses measured using AFM. In addition, in order to verify the authenticity of the colors we observed with the naked eye, the reflectance spectral intensity in the visible (380–780 nm) range was measured using a UV-visible NIR spectrophotometer and marked in the CIE1931 standard colorimetric card. In contrast to traditional colors generated by chemical dyes or pigments through selective absorption/reflection of light, structural colors produced by the interaction between light and the nanostructure strongly depend on the shape of the nanostructure. By this method, we were able to establish a correlation between sputtering time, film thickness, and color. Therefore, by combining UV lithography technology with magnetron sputtering of metal thin films, we ultimately created nanoart on a silicon substrate. Figure 1l–n shows our self-designed nanoart works, which were patterned with UV lithography and colorized with sputtering Cr metal.

The experimental results in Fig. S2a and b revealed the correlation between sputtering time, nanofilm thickness, and the corresponding nanofilm colors. Thus, we were able to control the nanofilm colors to our needs. Figure 1l depicts ‘Prometheus Stealing Fire,’ vividly illustrating the ancient Greek myth of Prometheus stealing fire from Apollo and gifting it to humanity, bringing light to the world. We can clearly see the facial features of the characters and the blazing torches in the picture. After chromium deposition, the artwork exhibited a distinct color contrast with the background, ultimately presenting a golden color (achieved with ∼50 nm chromium film). Figure 1m and n showcase a green pastoral landscape (∼30 nm chromium film) and a blue university crest (∼17 nm chromium film), respectively.

In addition to presenting a single color on the entire silicon wafer, as mentioned above, multiple colors can also be obtained through other means. For instance, prior to metal deposition, plasma etching can be employed to destroy the uniformity of the silicon surface, creating a rough texture [10]. By controlling the thickness of the metal film, its light absorption and reflection properties can be altered. Additionally, multilayer film design can achieve a richer color by utilizing the different absorption/reflection properties of light. After undergoing lithography and magnetron sputtering coloring, the silicon wafers (∼1.2 cm square) are then sealed and packaged, ready for market distribution. This method can significantly reduce the cost of nanoart creation, making large-scale nanoart production feasible and enabling widespread circulation of the final products. We believe that the idea presented in this paper can serve as a medium with a relatively low technical barrier, allowing more people to understand and participate in nanoart creation.

## Supplementary Material

nwaf468_Supplemental_File
