# Supplementary material for: Nanotechnology opens up a new universe of nanoart
Source: Natl Sci Rev. 2025 Nov 4;12(12):nwaf468. doi: 10.1093/nsr/nwaf468 (PMC12704097; doi:10.1093/nsr/nwaf468)
Supplement: nwaf468_Supplemental_File [file nwaf468_supplemental_file.pdf]

Supplementary Data

Nanotechnology opens up a new nanoart universal

Jinsai Tian[a], Jiayi Chen[a], Yue Liu[a], Baokang Niu[a], Junhui Chen[a], Mengyu Yan[a, *], Kui Su[b, *]

[a]State Key Laboratory of Advanced Technology for Materials Synthesis and Processing, Wuhan University of Technology, Wuhan 430070, China

[b]International Students Office of Fudan University, Shanghai 200433 , China

*Corresponding author.

E-mail: ymy@whut.edu.cn; kui_su@fudan.edu.cn

Supplementary data include the following files:

Material and methods
Supplementary Figure 1
Supplementary Figure 2

Materials and Methods

Spin coat the cleaned Si/SiO_2 chip (285 nm oxide layer) with LOR3A and S1805 photoresist. The rotation speed is 2000 revolutions per minute, with a duration of 40 seconds. LOR3A photoresist was rotated on the wafer and heated at 160 ℃ for 5 minutes, while S1805 was heated at 100 ℃ for 1 minute. Then, the desired pattern can be obtained by UV lithography (TTT-07-UV Litho ACA PRO) and development processing. The photolithographed silicon wafer is then placed into a magnetron sputtering device (PD-200 C), where chromium metal was used as the evaporation source to sputter onto the photoresist surface. In this experiment, the sputtering power and argon gas flow rate were fixed, with sputtering time serving as the single variable. Finally, soak the sputtered silicon wafer in acetone and remove the residual glue to obtain nano art works of different colors.

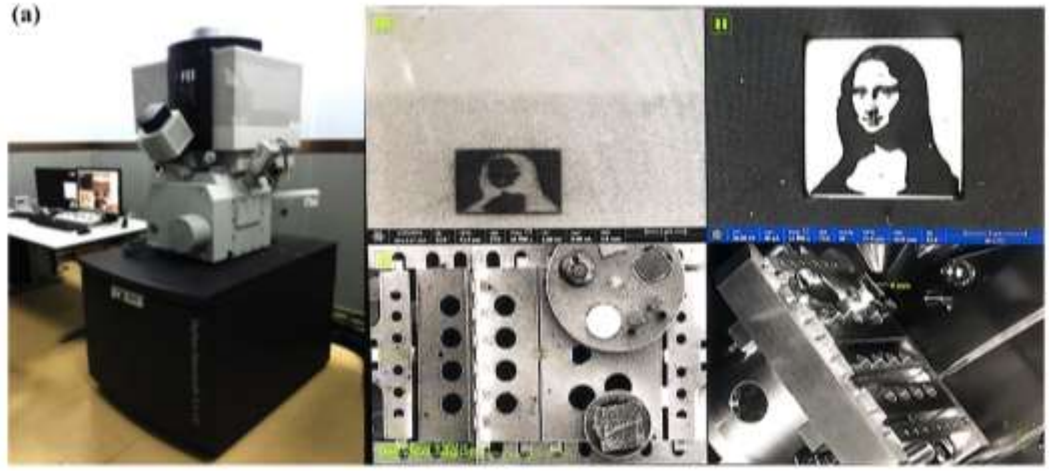

Supplementary Figure S1 Partial process of processing Mona Lisa image using focused ion beam.

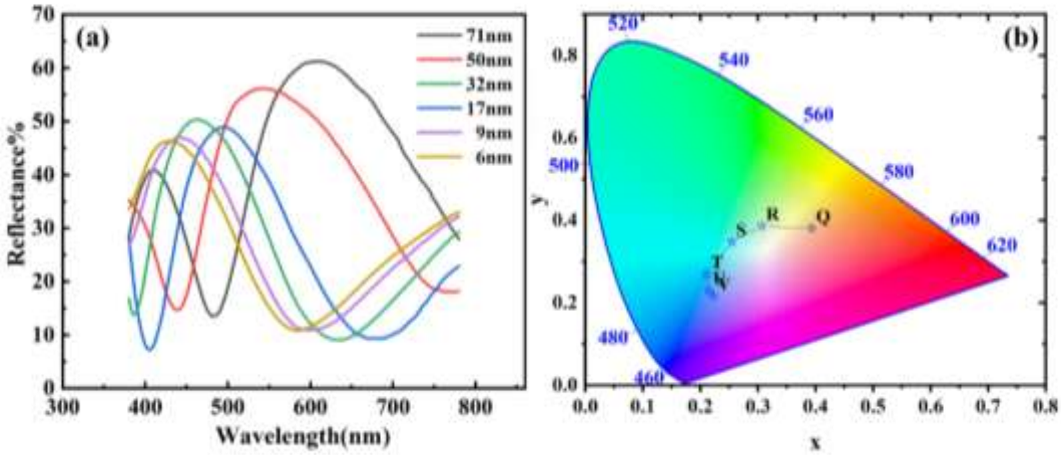

Supplementary Figure S2 Thickness-dependent reflection spectra of metal chromium films and their corresponding standard colors in CIE.
